# Implication of IL-17 in Bone Loss and Structural Damage in Inflammatory Rheumatic Diseases

**DOI:** 10.1155/2019/8659302

**Published:** 2019-08-14

**Authors:** Benoit Le Goff, Béatrice Bouvard, Thierry Lequerre, Eric Lespessailles, Hubert Marotte, Yves-Marie Pers, Bernard Cortet

**Affiliations:** ^1^Service de Rhumatologie, Hôtel-Dieu, 1 Place Alexis Ricordeau, 44093 Nantes Cedex 1, France; ^2^INSERM UMR 1238 Phy-OS, 1 Rue Gaston Veil, 44035 Nantes Cedex 1, France; ^3^Service de Rhumatologie, CHU d'Angers, 4 Rue Larrey, 49933 Angers Cedex 9, France; ^4^Groupe Etudes Remodelage Osseux et bioMatériaux, GEROM, EA 4658, SFR 4208, UNIV Angers, IRIS-IBS Institut de Biologie en Santé, CHU d'Angers, 49933 Angers, France; ^5^Service de Rhumatologie, CHU de Rouen, 1 Rue de Germont, 76031 Rouen Cedex, France; ^6^Département de Rhumatologie, CHR d'Orléans, EA 4709 I3MTO, Université d'Orléans, Orléans, France; ^7^SAINBIOSE, INSERM U1059, University of Lyon, Saint-Etienne F-42023, France; ^8^Service de Rhumatologie, CHU de Saint-Etienne, Avenue Albert Raimond, 42270 Saint-Etienne, France; ^9^IRMB, University Montpellier, INSERM, CHU Montpellier, Montpellier, France; ^10^Clinical Immunology and Osteoarticular Diseases Therapeutic Unit, Department of Rheumatology, Lapeyronie University Hospital, Montpellier, France; ^11^Bernard Cortet, EA 4490, Service de Rhumatologie, CHU Lille, Université Lille, 59000 Lille, France

## Abstract

Proinflammatory cytokines play an important role in the systemic and focal bone loss associated with chronic inflammatory diseases. Targeting these cytokines with biologics and small molecules has led to a major improvement of the bone health of patients with inflammatory arthritis. Cytokines from the IL-17 family have been shown to be involved in the pathogenesis of several diseases such as spondyloarthritis, psoriatic arthritis, or psoriasis. IL-17A has been the first described and the most studied. The recent development of targeted therapies against IL-17A or its receptor and their efficacy has confirmed the importance of this cytokine in the development of inflammatory diseases. The aim of this review was to describe the effects of the IL-17 family and more particularly of IL-17A on bone and cartilage tissues. At the cellular level, IL-17A is proosteoclastogenic whereas its effects on osteoblasts depend on the stage of differentiation of these cells. *In vivo*, IL-17A is not required for normal bone homeostasis but plays an important role in bone loss notably in an ovariectomized mouse model of osteoporosis. Preliminary data from clinical trials showed a stabilisation of bone density in patients treated with anti-IL-17A antibodies. IL-17A plays a central role in the cartilage damage through the induction of collagenases and by decreasing the expression of their inhibitors in synergy with the other proinflammatory cytokines. The prevention of structural damage by anti-IL-17A therapies has been demonstrated in several pivotal clinical trials. Overall, blocking the IL-17A pathway seems to have a positive effect on the bone and cartilage damage observed in inflammatory arthritis. Differences and specificity of these effects compared to those already described with other biologics such as anti-TNF therapies remain to be explored.

## 1. Introduction

The interleukin 17 (IL-17) family is composed of 6 cytokines (IL-17A to F) that play crucial roles in antimicrobial response and in the development of inflammatory diseases. The most widely investigated cytokine of this family, IL-17A, was discovered in 1993 [1]. IL-17F appears to be the other dominant proinflammatory cytokine of the IL-17 family [2]. IL-17 receptors (IL-17R) are expressed in almost every cell type, including epithelial cells, endothelial cells, fibroblasts, and myeloid cells, showing that IL-17 can act on diverse tissues throughout the body [3]. IL-17A signals through IL-17RA that is ubiquitously expressed on a wide range of tissues and cell types. Upon stimulation with IL-17A, IL-17RA initiates the activation of downstream signaling pathways to induce the production of proinflammatory molecules. However, IL-17RA alone is insufficient to mediate IL-17signaling. Further evaluation revealed that IL-17 signals through a heterodimeric receptor complex composed of IL-17RA and IL-17RC [4]. Th17 cells are the main producing cells of IL-17A, but other cells have been described as potential sources: natural killer (NK) cells, mast cells, neutrophils, and innate lymphoid cells [5, 6]. IL-17A is a prototypic proinflammatory cytokine. For instance, in the context of arthritis, IL-17A is able to induce IL-6 production by synoviocytes in synergy with the other proinflammatory cytokines such as IL-1 or TNF-alpha [7]. The effect of IL-17A on the immune system has already been reviewed elsewhere and is not the aim of this article [8].

The discovery of the IL-23-IL-17 immune pathway has led to major changes in the understanding of mechanisms leading to the development of autoimmune disease. Polarization of CD4 naïve cells towards Th17 cells under the influence of IL-23, but also TGF*β* and IL-6, has been shown to play an important role in several inflammatory diseases [9, 10]. The IL-17/IL-23 axis has been shown to be involved in the pathogenesis of spondyloarthropathies. A genetic association of ankylosing spondylitis with some IL-23R polymorphisms has been demonstrated in several studies [11]. IL-17 and IL-23 are elevated in the serum of patients with ankylosing spondylitis (AS) [12–14]. Animal models have confirmed that IL-23 overexpression induced axial and peripheral enthesitis and that IL-17 blockade significantly reduced disease severity [15]. These results led to the development and approval of drugs targeting IL-17 in AS, psoriasis, and psoriatic arthritis (PsA). Systemic bone loss, ankylosis, and joint destruction are some of the most frequent and severe complications of spondyloarthropathies. In AS, chronic joint inflammation might lead to ectopic new bone formation and a progressive ankylosis of the spine and sacroiliac joints. As in rheumatoid arthritis (RA), some patients with PsA develop severe peripheral joint destruction and disability [16]. Finally, chronic inflammation is associated with systemic osteoporosis and an increased risk of fragility fractures [17]. If the effect of IL-17A blocking therapies on pain and inflammation has been demonstrated in pivotal clinical trials, their effects on bone and on structural damage remain to be more thoroughly explored, especially in AS [18].

This review will focus on the effects of the IL-17 cytokine family on bone and cartilage tissues in the context of inflammatory arthritis. The availability of new drugs targeting the IL-17/IL-23 axis and the importance of structural damage in these diseases prompted us to review the main effect of these cytokines *in vitro* as in animal models of osteoporosis and arthritis. The relevance of these results will also be discussed in light of the data available from recent clinical trials.

## 2. IL-17 Effects on Bone

### 2.1. Effect of IL-17A on Bone Cells *In Vitro* (Figure 1)

#### 2.1.1. Osteoclasts

Osteoclasts are multinuclear cells derived from monocytic lineage. Receptor activator of nuclear factor kappa-B ligand (RANK-L) and macrophage colony-stimulating factor (M-CSF) are the master cytokines involved in the control of osteoclast differentiation. In inflammatory conditions, osteoclastogenesis can also be induced or enhanced by proinflammatory cytokines such as TNF-alpha, IL-1, and IL-6 that directly or indirectly promote osteoclast differentiation [19]. Kotake et al. have first demonstrated that IL-17 present in the synovial fluid (SF) from RA patients was a potent inductor of osteoclastogenesis [20]. Indeed, an anti-IL-17A antibody was able to inhibit the proosteoclastogenic effect of SF on osteoclast precursors. In a coculture model (osteoblast-osteoclast), they showed that this effect was indirect as osteoprotegerin (OPG) dose-dependently inhibited IL-17A-induced osteoclastogenesis. They concluded that IL-17A had a potent indirect effect on osteoclastogenesis through the stimulation of RANK-L expression by the osteoblasts.

A potential direct effect of IL-17A on osteoclasts remains a matter of debate. Some authors report that RANK-L was needed to observe a significant effect of IL-17A on osteoclastogenesis [21]. In this work, IL-17A was able to increase M-CSF-R and RANK expression on these precursors and therefore their response to M-CSF and RANK-L stimulation. However, IL-17 alone has no effect on osteoclastogenesis. On the other hand, other studies demonstrated a direct effect of IL-17A on osteoclast differentiation whereas Balani et al. and Yago et al. showed no effect of IL-17A on osteoclast development even in the presence of M-CSF and RANK-L [22, 23]. A recent work might give explanations for understanding these discrepancies [24]. The authors used different monocyte subtypes as a source for osteoclast precursors. Osteoclastogenesis and bone resorption by osteoclasts derived from classical monocytes remained unaffected by IL-17A, while osteoclast formation from intermediate monocytes was inhibited by the cytokine. Limited numbers of osteoclasts were formed from nonclassical monocytes on the bone, and no bone resorption was detected. This study showed that osteoclast number, size, nucleus number, and resorption activity were dependent on the type of monocytes used as the source of osteoclast precursors.

Overall, it seems clear that IL-17A has a strong indirect proosteoclastogenic effect mediated by RANK-L expression on osteoblasts. On the other hand, a potential direct effect of this cytokine remains debated and depends on the model and the subtype of cells used as a source for osteoclast precursors.

#### 2.1.2. Osteoblasts

Mesenchymal stem cells (MSCs) are multipotent stromal cells that can differentiate in specialized cells such as osteoblasts, adipocytes, chondrocytes, or myocytes depending on the environment and stimulation. Human MSCs (hMSCs) express the five members of the IL-17 receptor family, and it has been shown that IL-17A, secreted primarily by Th17 cells, can induce their proliferation and differentiation. Thus, Huang et al., using recombinant human IL-17A and primary hMSC isolated from patients' femur samples obtained during surgery, demonstrated that IL-17 (50 ng/ml) can stimulate the proliferation of hMSC in a manner dependent on the generation of reactive oxygen species (ROS) [25]. IL-17A also induced the motility and migration of hMSCs and their differentiation in osteoblasts, increasing the expression of alkaline phosphatase and enhancing mineralization. IL-17 (50 ng/ml) is as potent as BMP-2 (30 ng/ml) in inducing osteogenic differentiation. Shin et al. showed that IL-17A significantly inhibited adipocyte differentiation in hMSCs and increased lipolysis of differentiated adipocytes [26]. Furthermore, IL-17A can significantly increase leptin production that inhibits adipogenesis and promotes osteogenesis on hMSCs via JAK/STAT signaling [27]. Finally, Osta et al. demonstrated that TNF-alpha and IL-17A could interact in a synergistic way to induce osteogenic differentiation of hMSCs [28].

In contrast with effects of IL-17A on hMSC, Kim et al. showed that IL-17A inhibits rat calvarial osteoblast precursor cells and bone regeneration in a rat model of calvarial defect [29]. Using murine calvarial osteoblasts from a K/BxN serum transfer murine model of inflammatory arthritis, Shaw et al. demonstrated that IL-17A inhibits osteoblast differentiation, inducing mRNA expression of the Wnt antagonist secreted frizzled-related protein 1 (sFRP1). A blocking antibody directed against sFRP1 reduced the inhibitory effect of IL-17A on differentiation [30].

Overall, IL-17A stimulates the proliferation of hMSCs and their differentiation in osteoblasts in humans. IL-17A might inhibit this differentiation when acting on more differentiated cells such as calvarial precursors in mice.

### 2.2. Role of IL-17A in Bone Remodeling: An *In Vivo* Approach (Table 1)

If the study of IL-17 effects *in vitro* is interesting to better understand its mode of action at the cellular level, an *in vivo* approach remains essential to capture its global effect on bone remodeling. IL-17A^−/−^-deficient mouse studies showed that IL-17A does not play a role in physiological bone remodeling: both the cortical and the trabecular bone in femurs of IL-17A-deficient and control C57BL/6 mice exhibit similar bone mineral density, trabecular bone volume (BV/TV), bone formation rate, and no increase in osteoclast number [31, 32]. On the other hand, IL-17A overexpression by a minicircle DNA led to a bone phenotype with periarticular bone loss mimicking an erosive inflammatory arthritis without arthritis evidence [21]. However, the authors did not give information regarding the effect of this overexpression on systemic bone loss.

In bone disease, the role of IL-17A on bone remodeling seems to be different. In ovariectomized mice, there was an increase in the number of Th17 cells, transcription factors promoting Th17 cell differentiation, and circulating IL-17A levels. These effects were reversed by oestrogen supplementation [33]. DeSelm et al. showed that mice lacking the principal IL-17 receptor or its effector, Act1, are protected from the skeletal effects of ovariectomy (OVX) [34]. Four weeks of oestrogen deficiency substantially diminished the trabecular bone in wild-type mice whereas those lacking IL-17RA were completely protected. Furthermore, the serum C-terminal telopeptide (CTX) was not elevated in mice lacking the receptor compared to wild-type mice. The administration of anti-human IL-17A mAb also prevented bone loss. Tyagi et al. investigated the effects of anti-TNF-alpha, anti-RANK-L, or anti-IL-17A antibody administration to oestrogen-deficient mice on skeletal parameters [35]. In this study, adult Balb/c mice were treated with anti-RANK-L, anti-TNF-alpha, or anti-IL-17A subcutaneously, twice a week for 4 weeks after ovariectomy. All three antibodies restored the trabecular microarchitecture with comparable efficacy; however, cortical bone parameters, bone biomechanical properties, and histomorphometry were best preserved by anti-IL-17A antibody. Also, they showed that all three antibodies equally inhibited the OVX-induced increase in sclerostin mRNA levels but anti-IL-17A antibody treatment induces the expression of T cell-produced Wnt10b, which then activates canonical Wnt signaling, leading to increased osteoblast differentiation and bone mass.

Overall, if IL-17A is not a major cytokine implicated in physiological bone remodeling, studies support an important role of this cytokine in pathological conditions. Interestingly, anti-IL-17A was as potent as an anti-RANK-L in the prevention of bone loss induced in mice after ovariectomy. As systemic bone loss is also part of the complication of chronic inflammatory arthritis, the question is to know if these promising results translate to human disease.

### 2.3. IL-17A, Bone Loss, and Effect of Anti-IL-17A Therapies on the Bone in Patients with Inflammatory Arthritis

Systemic osteoporosis and increased risk of fragility fractures are associated with inflammatory chronic rheumatisms. Although the mechanisms explaining this relationship are not fully elucidated, T cells play a major role in the cross-talk between the immune system and the bone [36, 37]. Various proinflammatory cytokines are involved in the bone remodeling process and consequently are implicated in osteoporosis [38]. In an elegant study, conducted both in mice and humans (psoriasis patients), levels of cytokines, bone biomarkers, and bone density and structure were assessed by high-resolution peripheral quantitative computed tomography at the metacarpal heads [39]. It was found that, compared to healthy controls, psoriasis patients exhibited lower trabecular number, bone volume per tissue volume (BV/TV), and trabecular BMD. In parallel, patients with measurable IL-17A serum levels had significantly lower BV/TV than those without detectable IL-17A. Compared to healthy controls, those with psoriasis had lower levels of osteocalcin and procollagen type 1 N-terminal propeptide indicating decreased bone formation. These data might indicate the following in psoriasis patients: first, a periarticular bone loss exists; second, this bone loss might be associated with a defective bone formation; and third, this bone loss is associated with the IL-17A levels. The role of IL-17 in focal bone involvement has been extensively studied [40]. However, prospective studies in humans exploring the potential systemic effect on the bone that could be associated with the use of anti-IL-17A agents in chronic inflammatory rheumatic diseases are limited. Nevertheless, recently, preliminary data were presented at the 2018 ACR meeting. This was a post hoc analysis of the pivotal MEASURE 1 study using secukinumab 150 mg/month [41]. 104 patients with AS were studied. Both BMD and bone turnover markers (BTM) were assessed at baseline and at weeks 52 and 104. The BTM assessed were osteocalcin, bone alkaline phosphatase (BAP), procollagen type 1 N-terminal propeptide (P1NP), and procollagen-1 carboxyterminal peptide (P1CP) for bone formation and CTX for bone resorption. The mean age of the population at baseline was 40.3 ± 12.3 years and 66% were male patients. At 52 weeks, there was a moderate increase of BMD at the lumbar spine (23 mg/cm^2^ of hydroxyapatite, i.e., +2.6%). The increase was more pronounced at 104 weeks (42 mg/cm^2^ of hydroxyapatite, i.e., +4.7%). At the total hip, results were stable over time: +0.9% and +0.5% at 52 and 104 weeks, respectively. Findings were almost the same at the femoral neck: +0.8% and +0.2% at 52 and 104 weeks, respectively. Levels of BTM did not change over time.

## 3. Role of IL-17A in Cartilage and Joint Biology

### 3.1. IL-17A and Cartilage: *In Vitro* Effects

Proinflammatory cytokines (IL-6, IL-1*β*, TNF-alpha, and IL-17A) are present in synovial fluid and play a central role in the cartilage damage [42]. Secreted by many cell types (neutrophils, mast cells, Th17, gamma-delta T cells, NKT, and ILC3), IL-17A promotes the production and releasing of other proinflammatory cytokines from chondrocytes and synovial fibroblasts [8]. Thus, IL-17A disrupts extracellular matrix (ECM) homeostasis both independently and synergistically with TNF-alpha, IL-6, IL-1*β* [43, 44], or adipokines [45]. Studies on the role of IL-17 in the articular environment have most often been conducted in subjects with rheumatoid arthritis or osteoarthritis (OA). The percentage of cells secreting IL-17A in synovial tissue and the frequency of chondrocytes expressing the IL-17 receptor appears quite similar in these two conditions [46]. IL-17A acts on chondrocytes by activating inducible nitric oxide synthase (iNOS) expression, COX2 expression, or IL-6 secretion which participates in cartilage degradation. Moreover, IL-17A inhibits proteoglycan synthesis, enhances NO production, and acts in synergy with TNF-alpha in the destruction of the cartilage matrix [47–49]. Other studies suggest that IL-17F stimulates cartilage degradation by increasing the expression of collagenases (MMP-1 and MMP-13) and stromelysin-1 (MMP-3) and by decreasing the expression of their inhibitors (TIMP-2 and TIMP-4) or ECM components (type II collagen, aggrecan) [50]. Furthermore, IL-17A stimulates the release of a few chemokines (CXCL1, IL-8, and CCL2) by human chondrocytes, which also induces the secretion of MMPs or iNOS. However, this function seems lower compared to IL-1*β* stimulation [46]. In addition, mesenchymal stromal cells (MSCs) can differentiate into different lineages, such as osteoblasts and chondrocytes. Under inflammatory conditions, IL-17A inhibits chondrogenesis derived from human MSCs by suppressing PKA (protein kinase A) activity and SOX9 phosphorylation [51]. All these elements highlight IL-17 family involvement on cartilage matrix homeostasis.

### 3.2. Role of IL-17A in Joint and Cartilage Destruction *In Vivo* (Table 1)

As previously described, systemic overexpression of IL-17A in mice induced a systemic inflammation associated with a phenotype of psoriasis and joint destruction by induction of osteoclastogenesis [21]. In this model, joint damage was observed independently of any local inflammation. IL-17A overexpression exacerbated synovial inflammation and bone loss in the collagen-induced arthritis (CIA) model. Injected mice initiated arthritis at much earlier time points as compared with controls. Adenoviral IL-17A injection in the knee joint of type II collagen-immunized mice accelerated the onset and aggravated the synovial inflammation at the site. An upregulation of the synovial RANK-L/OPG ratio and an enhanced osteoclastic activity were observed [52]. Interestingly, blocking of IL-1 in this model with neutralizing Abs had no effect on the IL-17A-induced inflammation and joint damage in the knee joint, implying an IL-1-independent pathway. On the other side, blocking of endogenous IL-17A in the CIA model results in suppression of arthritis and reduction of joint damage. This preventive effect was also observed in mice after an IL-17A-deficient allogeneic bone marrow.

### 3.3. Structural Effect of Anti-IL-17A Therapies

RA and PsA share common clinical, radiological, and health-related quality of life characteristics. Contrary to the general idea, PsA may be just as destructive as RA [53]. Indeed, 47% of PsA patients present at least one erosion within two years of disease onset, and 67% of patients followed in PsA clinics have an erosive disease, inducing deformities and reducing the quality of life [16]. Moreover, among the patients having polyarticular PsA, at least 20% are at risk of progressing to a severe destructive phenotype (mutilans) comparable with that observed in RA.

In PsA, while methotrexate and sulfasalazine were unable to stop structural progression, TNF-alpha blocking agents as well as ustekinumab and secukinumab decrease the progression of structural damage [54–56]. Eder et al. showed that methotrexate increased the risk of structural progression much more than the TNF-alpha blocking agent [57]. Moreover, a meta-analysis performed from five studies focused on structural progression showed that the TNF-alpha blocking agent decreases the structural progression compared to placebo [55].

In the phase III, double-blind, placebo-controlled FUTURE 1 study, 606 PsA patients were randomized to intravenously receive secukinumab (10 mg/kg at weeks 0, 2, and 4) followed subcutaneously by secukinumab (75 or 150 mg every 4 weeks) or placebo [58]. van der Heijde showed that secukinumab inhibited radiographic progression at 52 weeks, with an effect on both erosion and joint space narrowing, irrespective of concomitant methotrexate use, and also in patients previously treated by TNF-alpha blocking agents. More than 80% of PsA patients had inhibition of progression from baseline to week 52. Compared with placebo, secukinumab demonstrated a disease-modifying effect even if the radiological progression was low in these patients and even if the clinical relevance of the reduction of radiographic progression is weak. In the FUTURE 5 clinical trial, including 996 patients with active psoriatic disease, receiving a high dose of secukinumab (150 or 300 mg) with or without a loading dose, or placebo (at 0, 1, 2, and 3 weeks and every 4 weeks), secukinumab reduced significantly radiographic structural progression compared to the placebo [59]. The proportion of patients with no radiographic structural progression was higher with secukinumab than with placebo at week 24. Moreover, the majority (85-92%) of secukinumab-treated patients showed no radiographic progression through 52 weeks of therapy. This study confirms the disease-modifying effect of secukinumab at both 300 and 150 mg doses with a loading dose. Moreover, the radiographic progression followed a similar trend in the subgroup of patients by prior anti-TNF therapy status through 52 weeks, with higher inhibition in the anti-TNF-naïve group than in patients with a previous inadequate response or intolerance to one anti-TNF.

Ixekizumab, another IL-17A inhibitor, was assessed in the SPIRIT-P1 phase 3 clinical trial in biologic DMARD-naïve patients with active psoriatic arthritis [60]. Patients were randomized to subcutaneous injections of placebo, adalimumab (40 mg every 2 weeks), ixekizumab 80 mg every 2 weeks, or ixekizumab 80 mg every 4 weeks after a 160 mg starting dose. As well as adalimumab, both regimens of ixekizumab were shown to be superior to placebo in inhibiting the progression of structural joint damage in patients treated for 24 weeks. The majority of the patients treated with ixekizumab had no radiographic disease progression over a period of 3 years [61]. All these data suggest a low mean progression of structural changes in PsA patients with IL-17 inhibitors.

## 4. Conclusion

Overall, IL-17A plays a major role in bone loss and cartilage damage associated with inflammatory arthritis. The effects on bone loss observed with anti-IL-17A therapies in animal models of postmenopausal osteoporosis raised the question of a specific effect of this cytokine on the bone compared to other proinflammatory cytokines such as TNF-alpha or IL-6. Preliminary data obtained from a clinical trial does not support this hypothesis for the moment as the effect observed in spondyloarthritis patients remains equivalent to those described previously with anti-TNF therapies. Several hypotheses can be raised to explain these discrepancies. First, although decrease in BMD is found in half of the patients with spondyloarthropathies, osteoporosis and vertebral fractures are actually rare. For instance, Malochet-Guinamand et al. recently found a prevalence of osteoporosis of 6.7% (according to WHO criteria) and vertebral fractures of 6.2% in 89 patients with a mean 10 years of disease evolution [62]. The effect of IL-17A on the bone has been studied in animal models of osteoporosis. Therefore, the effect of the IL-17A blocking agent on the bone would be more relevant to study in patients with postmenopausal osteoporosis or in a subgroup of patients with significant bone loss. Second, new advances in the understanding of bone remodeling in spondyloarthropathies has shown that other factors other than inflammation (mechanical stress, microbiota) can play a major role in the development of the bone loss phenotype observed in patients [63]. Finally, translating data from mice to human is always challenging. More studies will be needed in the future to answer this question. In the same line, the structural effect observed in patients might be due to an anti-inflammation more than a specific effect on cartilage. More studies of the role of IL-17A in diseases such as osteoarthritis, where cartilage damage is at the center of the pathophysiology, remain to be undertaken. A potential clinical effect of IL-17A blocking therapies in osteoarthritis will also be an interesting topic to explore.

## Figures and Tables

**Figure 1:**
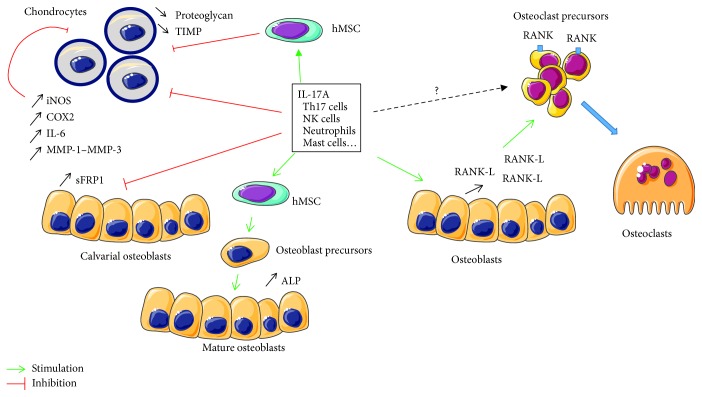
Summary of the *in vitro* effects of IL-17 on osteoblast, osteoclast, and chondrocyte differentiation. RANK-L: receptor activator of nuclear factor kappa-B ligand; RANK: receptor activator of nuclear factor kappa-B; hMSC: human mesenchymal stem cells; TIMP: tissue inhibitors of metalloproteinases; iNOS: inducible nitric oxide synthase; COX2: cyclooxygenase-2; MMP: matrix metalloproteinase; sFRP1: secreted frizzled-related protein 1; ALP: alkaline phosphatase.

**Table 1:** Summary of the main findings of IL-17 implication in bone loss and structural damage in animal models.

References	Animal	Animal model	Treatment/intervention	Main results
[22, 23]	IL-17A^−/−^ mice C57BL/6J mice	NA		IL-17A^−/−^ mice have physiological bone homeostasis indistinguishable from Wnt mice
[14]	IL-17 overexpression C57BL/6J mice		Systemic recombinant minicircle injection	Peripheral joint destruction without joint inflammation but some psoriasis Systemic bone loss not studied
	IL-17 overexpression C57BL/6J mice	Collagen-induced arthritis	Systemic recombinant minicircle injection	IL-17A exacerbates synovial inflammation and bone loss in inflammatory arthritis
[25]	IL-17RA^−/−^ mice	Ovariectomy	NA	Protected against bone loss
[24]	Adult Balb/c mice	Ovariectomy	Anti-IL-17 antibody	Prevented the bone loss phenotypes
[26]	Adult Balb/c mice	Ovariectomy	Anti-IL-17, anti-TNF, and anti-RANK-L antibodies	Cortical bone parameters, bone biomechanical properties, and histomorphometry were best preserved by anti-IL-17 antibody
[55]	Balb/c mice	Defects in middiaphysis region of femur	Anti-IL-17 antibody	Improvement of bone healing by recruitment of osteoprogenitors at the injury site
[20]	Sprague-Dawley rats	Critical-sized defects in the calvaria	Defects were filled with IL-17	IL-17 significantly inhibited the filling of calvarial defects *in vivo*
[43]	DBA/1 mice	Collagen-induced arthritis	Adenoviral IL-17 injected in the knee joint	Accelerated the onset and aggravated the synovial inflammation at the site, enhanced osteoclastic activity
[45]	DBA/1 mice	Collagen-induced arthritis	IL-17-deficient allogeneic bone marrow transplantation	Decreased arthritis severity associated with increased functionally suppressive Tregs and reduced levels of other Th17-lineage inflammatory cytokines
[44]	DBA/1 mice	Collagen-induced arthritis	Anti-IL-17 antibody	Treatment even after the onset of CIA significantly reduced the severity of CIA and suppressed joint damage

## Additional Points

*Highlights*. IL-17 plays a major role in bone loss and cartilage damage associated with inflammatory arthritis. The effects on the bone observed with anti-IL-17 therapies in animal models raise the question of a specific effect of this cytokine on the bone. Preliminary data obtained from a clinical trial does not support this hypothesis for the moment, and more studies will be needed to answer this question.
